# The phased telomere-to-telomere reference genome of *Musa acuminata*, a main contributor to banana cultivars

**DOI:** 10.1038/s41597-023-02546-9

**Published:** 2023-09-16

**Authors:** Xin Liu, Rida Arshad, Xu Wang, Wei-Ming Li, Yongfeng Zhou, Xue-Jun Ge, Hui-Run Huang

**Affiliations:** 1 Key Laboratory of Plant Resources Conservation and Sustainable Utilization, South China Botanical Garden, Chinese Academy of Sciences, Guangzhou, 510650 China; 2 South China National Botanical Garden, Guangzhou, 510650 China; 3 University of Chinese Academy of Sciences, Beijing, 100049 China; 4 National Key Laboratory of Tropical Crop Breeding, Shenzhen Branch, Guangdong Laboratory of Lingnan Modern Agriculture, Key Laboratory of Synthetic Biology, Ministry of Agriculture and Rural Affairs, Agricultural Genomics Institute at Shenzhen, Chinese Academy of Agricultural Sciences, Shenzhen, 518120 China; 5 School of Marine Sciences and Biotechnology, Guangxi University for Nationalities, Nanning, 530008 China; 6 National Key Laboratory of Tropical Crop Breeding, Tropical Crops Genetic Resources Institute, Chinese Academy of Tropical Agricultural Sciences, Haikou, 571101 China

**Keywords:** Structural variation, Comparative genomics, DNA sequencing, Natural variation in plants

## Abstract

*Musa acuminata* is a main wild contributor to banana cultivars. Here, we reported a haplotype-resolved and telomere-to-telomere reference genome of *M. acuminata* by incorporating PacBio HiFi reads, Nanopore ultra-long reads, and Hi-C data. The genome size of the two haploid assemblies was estimated to be 469.83 Mb and 470.21 Mb, respectively. Multiple assessments confirmed the contiguity (contig N50: 16.53 Mb and 18.58 Mb; LAI: 20.18 and 19.48), completeness (BUSCOs: 98.57% and 98.57%), and correctness (QV: 45.97 and 46.12) of the genome. The repetitive sequences accounted for about half of the genome size. In total, 40,889 and 38,269 protein-coding genes were annotated in the two haploid assemblies, respectively, of which 9.56% and 3.37% were newly predicted. Genome comparison identified a large reciprocal translocation involving 3 Mb and 10 Mb from chromosomes 01 and 04 within *M. acuminata*. This reference genome of *M. acuminata* provides a valuable resource for further understanding of subgenome evolution of *Musa* species, and precise genetic improvement of banana.

## Background & Summary

The wild relatives of domesticated crops, i.e. crop wild relatives (CWRs), generally possess genetic diversity helpful in developing more productive and resilient crop varieties, thereby providing a wide practical gene pool for genetic improvement of crops^1^. In order to address the challenges and threats posed by emerging diseases and climate change, CWRs appear to be a source for solutions to manage both biotic and abiotic stresses^2,3^. At present, combining huge sequence information and precise gene-editing tools provides a route to transform CWRs into ideal crops^2^. Therefore, a high-quality reference genome of CWR germplasm is an important prerequisite for efficiently introducing potential useful genes into breeding programmes. Thanks to the advances in sequencing technologies and analytical tools, many high-quality reference genomes for crops as well as their important wild relatives have been generated. These genetic resources will thus facilitate the identification of structural variants and incorporation of the variants from CWRs into crop gene pools.

Banana domestication started at least 7000 years ago in Southeast Asia^4^. Hybridization between various species and subspecies of the *Musa* genus led to the development of modern bananas with high production^5^. To date, most banana cultivars were derived from *Musa acuminata* (A genome), a complex of subspecies geographically segregated in distinct Southeast Asian continental regions and islands^6^. Four particular *M*. *acuminata* subspecies have been raised as the main contributors of edible banana cultivars, which are *banksii*, *burmannica*, *malaccensis*, and *zebrina*^4^. Several large structural variants in these subspecies were identified and suggested to be associated with the domestication of banana^7–11^. Genome research first started in the subspecies *malaccensis*. The first draft genome of *M*. *acuminata* ssp. *malaccensis* was assembled by incoporating Sanger and Roche/454 reads, with sequence errors corrected by Illumina data^12^. This assembly was anchored along the *Musa* linkage groups of the genetic map built with SSR and DArT markers. The double-haploid genotype (DH-Pahang) was used in this study for reducing genome complexity and facilitating assembly process. Recently the telomere-to-telomere (T2T) reference genome of DH-Pahang has been constructed using Nanopore data and polished with Nanopore and Illumina reads, with continuity improved significantly^13^. Although DH genotype could miss some important genetic information, these genome resources have significantly facilitated the studies of banana domestication and genome evolution. With advances in the sequencing technologies and biosoftwares, heterozygosity would not be the consistent hurdle. Currently, more and more haplotype-resolved and T2T genomes have been published, such as lychee^14^ and apple^15^, providing unprecedented insights into subgenome evolution and domesticated history.

**Fig. 1:**
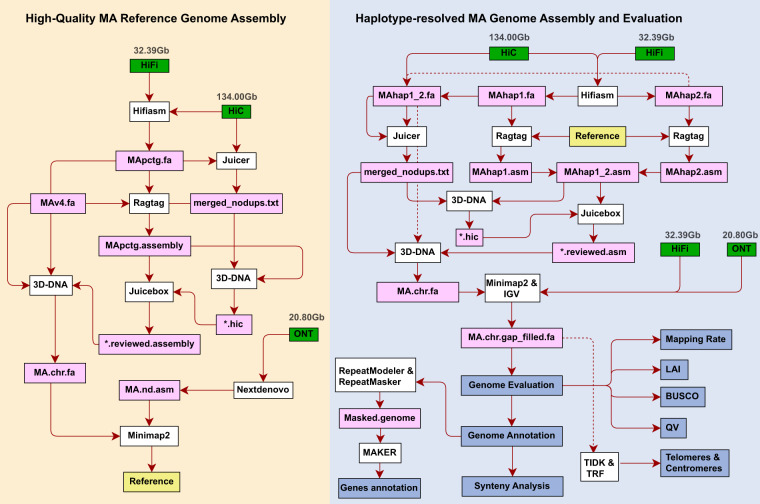
The workflow of generating haplotype-resolved and telomere-to-telomere reference genome for *M. acuminata*. The unphased reference genome was constructed following the workflow on the left yellow panel. Then a haplotype-resolved and telomere-to-telomere reference genome was produced according to the scheme on the right blue panel. Green boxes represent raw sequencing data; white boxes represent the tools used in this pipeline; pink boxes represent intermediate data; blue boxes represent post-analysis process.

In this study, we assembled a haplotype-resolved and telomere-to-telomere reference genome of *M*. *acuminata* ssp. *malaccensis* by incorporating PacBio HiFi reads, Nanopore ultra-long reads, and high throughput chromatin conformation capture (Hi-C) paired reads. An unphased reference genome was first constructed and used for guiding haplotype-resolved scaffolding (Fig. 1). Multiple assessment methods were applied to evaluate the quality of the haplotype-resolved assembly. A comprehensive genome comparison between this assembly and the previous reference of the DH genotype identified a large reciprocal translocation involving 3 Mb and 10 Mb from chromosomes 01 and 04. Furthermore, the 3-Mb segment (34,734,628 to 37,810,715 bp in chromosome 04) was suggested to be associated with flower development pathway, such as anther/stamen development. The haplotype-resolved genome of *M. acuminata* will help to obtain a better understanding of potential structural variants, allele specific expression and subgenome evolution of *Musa* species, and serve as reliable reference for banana breeding programmes.

## Methods

### Sample collection and sequencing

The *M*. *acuminata* sample used for DNA and RNA extraction was obtained from South China Botanical Garden, Chinese Academy of Sciences, Guangzhou, China. Tissues were immediately frozen in liquid nitrogen and preserved at −80 °C for DNA/RNA extraction. The CTAB method was used to extract high quality genomic DNA from leaf tissue samples.

A standard SMRTbell library was constructed using SMRTbell Express Template Prep Kit 2.0 according to the manufacturer’s recommendations (Pacific Biosciences, CA, USA) and sequenced on a PacBio Sequel II platform. This yielded 32.39 Gb HiFi data, covering ~65 × coverage of the haploid genome size. The N50 length of the HiFi reads was 17.32 kb. A nanopore library was constructed with the Oxford Nanopore SQK-LSK109 kit following the manufacturers’ instructions and sequenced on a PromethION platform. Totally 20.80 Gb ONT data were obtained, covering ~42 × coverage of the haploid genome size. The N50 length was 86.86 kb. A Hi-C library was constructed based on cross-linked genomic DNA and sequenced on an Illumina NovaSeq platform (Illumina, San Diego, CA, USA). In total, 134 Gb Hi-C data were obtained, covering ~268 × coverage of the haploid genome size. The 15.58 Gb NGS data were obtained using the Illumina NovaSeq platform, covering ~31 × coverage of the haploid genome size (Table 1).

**Table 1:** Summary of sequencing data of *Musa acuminata* ssp. *malaccensis* for haplotype-resolved and telomere-to-telomere assembly and genome annotation.

Sequencing	Clean base (Gb)	Clean reads	N50 length (bp)	Depth (X)	Sample	Application
HiFi	32.39	1,793,624	17,320	64.78	Leaf	Assembly
HiC	134.00	894,989,890	2 × 150	268	Leaf	Chromosome construction
ONT	20.80	439,578	86,861	41.6	Leaf	Gap filling
Illumina	15.58	\	2 × 150	31.16	Leaf	Genome evaluation
RNA-seq	6.6	\	2 × 150	\	Flower	Genome annotation
RNA-seq	7.1	\	2 × 150	\	Fruit	Genome annotation
RNA-seq	6.1	\	2 × 150	\	Leaf	Genome annotation
RNA-seq	7.1	\	2 × 150	\	Root	Genome annotation

Additionally, total RNA was extracted from four tissues, including root, leaf, flower, and fruit, using the NEBNext^®^ Ultra^™^ II Directional RNA Library Prep Kit for Illumina^®^ (New England Biolabs, MA, USA). Paired-end 150-bp reads were also generated by the Illumina NovaSeq platform. These yielded a total of 26.90 Gb raw RNAseq data (Table 1). All sequencing were carried out at Anhui Double Helix Gene Technology Co., Ltd. (Anhui, China).

### Genome size and heterozygosity estimation

**Fig. 2:**
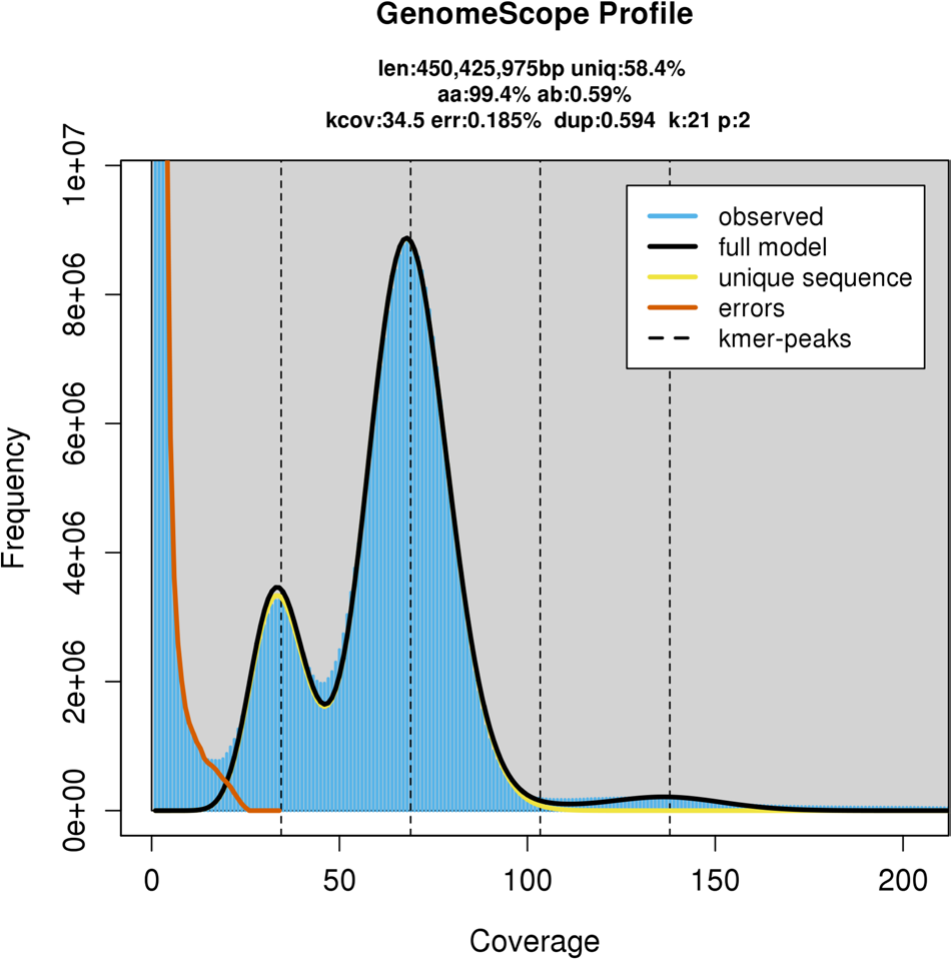
The GenomeScope profile of *M. acuminata* based on 21 K-mer.

CCS software (https://github.com/PacificBiosciences/ccs) with default parameters was used to generate the consensus reads (HiFi reads). Based on the obtained high-accurate HiFi reads, the K-mer distribution was analysed with jellyfish^16^ with jellyfish count -C -m 21 -s 100000000 and jellyfish histo -h 1000000. The results were subsequently imported to GenomeScope v2.0^17^ with K-mer length = 21 and Ploidy = 2. The genome size of *M*. *acuminata* was estimated to be 450.43 Mb with the 21 K-mer, about 14% shorter than DH-Pahang genome size (523.00 Mb) estimated by flow cytometry^12^. The heterozygosity rate was estimated to be 0.59% (Fig. 2).

### *De novo* haplotype-resolved genome assembly

Fastp v0.23.2^18^ was performed to filter Hi-C reads with default parameters. Subsequently, hifiasm v0.16.1-r375^19^ was carried out to generate the primary unphased draft genome based on HiFi and Hi-C reads. This generated a 491.54 Mb draft genome with an N50 of 26.62 Mb, and only 20 contigs consisted of 90% length of the genome (Table 2). Then, ragtag v2.1.0^20^ with default parameters was first used to sort, orientate, and cluster the primary contigs guided by the T2T version of *M*. *acuminata* ssp. *malaccensis* DH-Pahang genome^13^ (Hereafter MAv4). Meanwhile, the primary contigs were anchored into 11 pseudo-chromosomes using Juicer v1.6^21^ and 3D-DNA v180922^22^ in turn. Then, based on the assembly file obtained from ragtag and the hic file from Juicer and 3D-DNA, Juicebox v2.20.00^23^ was introduced for visualizing Hi-C data and manual correction in order to obtain a high-quality reference genome. Finally, there were only 17 gaps in the high-quality reference genome. For gap filling, ONT assembly was constructed by NextDenovo (https://github.com/Nextomics/NextDenovo) with read-cutoff = 1k and genome_size = 500 M. Then this draft ONT assembly was polished by Nextpolish^24^ based on the HiFi reads and the Illumina reads with default parameters. Subsequently minimap2 v2.24-r1122^25^ with default parameters was used to map the polished ONT assembly to the primary reference genome. We examined the breakpoint with the Integrative Genomics Viewer (IGV) tool^26^ and manually filled the gaps based on the alignment results. After using ONT assembly to fill all remaining gaps, a high-quality reference genome named MA was generated. The genome size of this unphased assembly is 471.04 Mb with an anchored rate of 95.83%. The Hi-C heatmap confirmed the contiguity of the assembly (Supplementary Figure S1).

**Table 2:** Summary of genome assembly of *Musa acuminata* ssp. *malaccensis* genome.

Assembly	MA	MAH1	MAH2
contigs (> = 0 bp)	141	275	206
contigs (> = 1000 bp)	141	275	206
contigs (> = 5000 bp)	141	275	206
contigs (> = 10000 bp)	141	275	206
contigs (> = 25000 bp)	132	235	194
contigs (> = 50000 bp)	112	180	139
Total length (> = 0 bp)	491,526,655	500,781,154	484,357,301
Total length (> = 1000 bp)	491,526,655	500,781,154	484,357,301
Total length (> = 5000 bp)	491,526,655	500,781,154	484,357,301
Total length (> = 10000 bp)	491,526,655	500,781,154	484,357,301
Total length (> = 25000 bp)	491,334,973	499,946,784	484,102,230
Total length (> = 50000 bp)	490,674,857	498,063,881	482,072,416
contigs	141	275	206
Largest contig	50,229,097	50,630,355	50,002,820
Total length	491,526,655	500,781,154	484,357,301
GC (%)	39.58	39.89	39.69
N50	26,620,819	16,527,116	18,582,139
N90	7,537,575	4,161,334	6,090,445
auN	26,629,771	20,028,603	19,951,612
L50	7	9	10
L90	20	33	28

Note: MA represents the primary contig sets, while MAH1 and MAH2 represent contigs in haplotype1 and contigs in haplotype2.

**Fig. 3:**
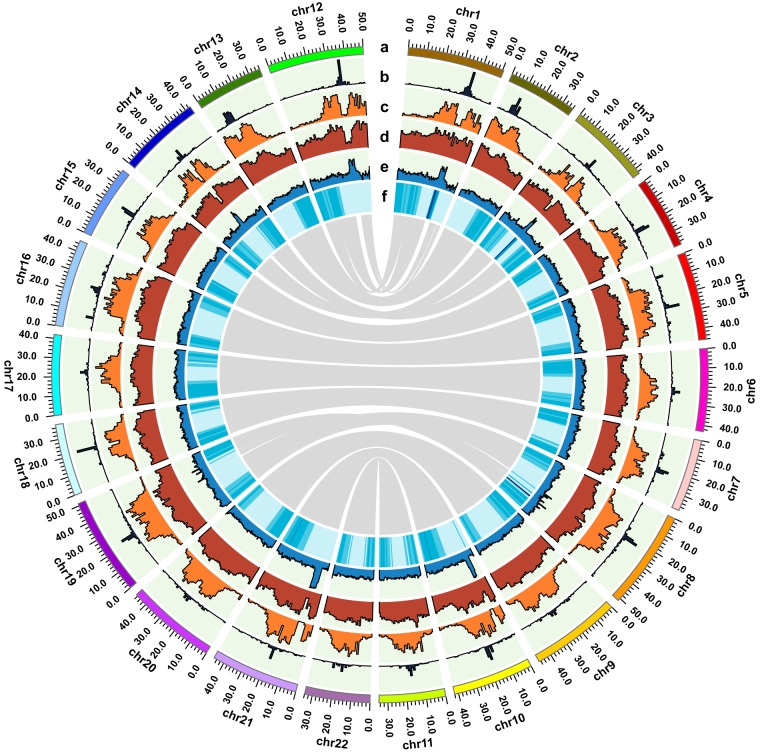
The Overview of *M. acuminata* genome assembly and features. The tracks represent the following elements (from outer to inner): (**a**) Karyotypes of the 22 chromosome sequences, (**b**) TRF-183bp centromeric repeat density, (**c**) *Copia* density, (**d**) Transposable element (TE) density, (**e**) GC contents, (**f**) Gene density. The innermost is syntenic relationships.

**Fig. 4:**
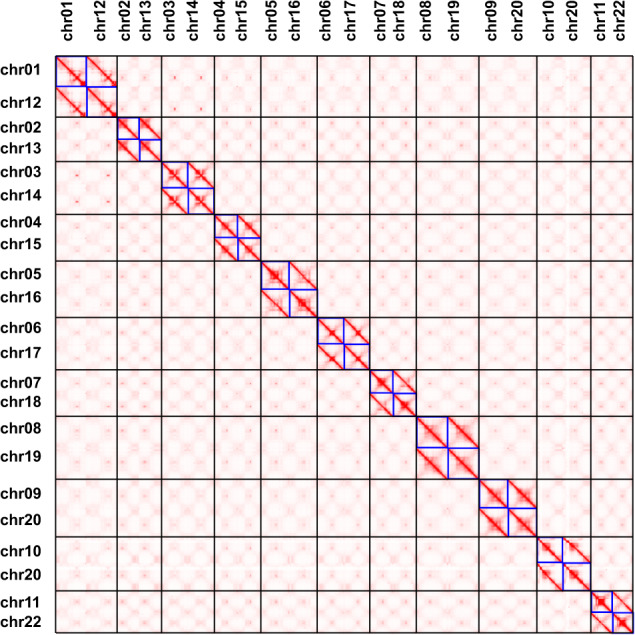
The Hi-C heatmap of haplotype-resolved genome of *M. acuminata*. 11 chromosome pairs were defined.

To obtain a haplotype-resolved genome, a similar pipeline was applied (Fig. 1). Two primary haploid assemblies were first generated by hifiasm. Further genome assembly statistics were performed with QUAST^27^ with default parameters. Accumulative lengths of the two haploid assemblies were 500.78 Mb and 484.36 Mb with N50 of 16.53 Mb and 18.58 Mb, respectively (Table 2). After Hi-C scaffolding processes, 469.83 Mb and 470.21 Mb were anchored to 11 chromosomes respectively, with an anchored rate of 93.82% and 97.08% (Table 3). The genome sizes of the two haploid assemblies were slightly longer than that of MAv4 (468.82 Mb)^13^, and represented approximately 90% of DH-Pahang genome size (523.00 Mb) estimated by flow cytometry^12^. All 66 gaps in the two haploid assemblies were filled. Finally, the haplotype-resolved and telomere-to-telomere reference genome for *M*. *acuminata* was obtained; and the two haploid assemblies were named MAH1 and MAH2. The circos^28^ software was introduced to draw the genome features shown in Fig. 3. The Hi-C heatmap confirmed this assembly as a complete and reliable haplotype-resolved reference genome (Fig. 4).

**Table 3:** The lengths of the pseudo-chromosomes of *Musa acuminata* ssp. *malaccensis* genomes.

CHR	MAH1		MAH2		MAv4	
	Length(bp)	Contigs	Length(bp)	Contigs	Length(bp)	Contigs
CHR01	50,630,355	1	50,002,820	1	41,765,374	7
CHR02	36,099,580	1	36,147,835	2	34,826,099	1
CHR03	43,044,357	4	42,983,428	4	43,931,233	4
CHR04	37,851,086	6	37,817,432	5	45,086,258	1
CHR05	46,163,207	7	45,655,733	9	46,513,039	4
CHR06	43,119,214	3	41,967,602	3	43,117,521	1
CHR07	37,893,627	6	37,753,859	3	39,373,400	5
CHR08	51,441,117	4	51,151,883	6	51,314,288	4
CHR09	46,958,856	3	47,015,202	3	47,719,527	1
CHR10	42,015,131	6	45,690,726	5	40,511,255	8
CHR11	34,610,164	3	34,028,409	3	34,663,808	1
Total	469,826,694	44	470,214,929	44	468,821,802	37

### Genome quality assessment

Multiple methods were combined to evaluate the quality of genome assembly. First, the HiFi, Illumina, and RNAseq reads were aligned to the phased genome using minimap2 v2.24-r1122, BWA v0.7.17-r1188^29^, and HiSAT2 v2.2.1^30^ with default parameters, respectively. BamTools v2.5.1^31^ was used to calculate the read mapping rates. The results showed a HiFi coverage rate of 99.86% and 99.87% on MAH1 and MAH2 assemblies, respectively. The mapping rate of Illumina reads reached up to 99.98% in both haploid assemblies. The mapping rate of RNAseq reads ranged from 92.44% to 97.34% (Table 4). Second, the LTR Assembly Index (LAI) calculated from LTR_retriever v2.9.0^32^ was used to assess the genome assembly quality. The LAI of MAH1 and MAH2 reached up to 20.18 and 19.48, respectively, indicating that our phased assembly reached the standard of a golden reference. Third, the completeness of the haplotype-resolved genome was evaluated by BUSCO v5.4.3^33^ against the ‘embryophyta_odb 10’ database. In total, 98.57% (1,591 of 1,614) of the complete BUSCO genes were identified (Table 5). Finally, the consensus quality value (QV) of the genome was assessed by Merqury v1.3^34^ with meryl k = 19 count, showing 45.97 and 46.12 of QV (Genome accuracy >99.99%) for MAH1 and MAH2, respectively (Table 6, Supplementary Figure S2).

**Table 4:** Assessment of genome quality based on mapping with RNAseq reads.

Tissues	Data size (Gb)	reads count	Mapping rate (%)	
			MAH1	MAH2
Flower	6.6	59145701	97.34	97.29
Fruit	7.1	52110912	97.19	97.17
Leaf	6.1	45400245	96.50	96.67
Root	7.1	58396498	92.47	92.44

**Table 5:** BUSCO results of MAH1 (C: 98.57%) and MAH2 (C: 98.57%).

	MAH1	MAH2
Complete BUSCOs (C)	1591	1591
Complete and single-copy BUSCOs (S)	1515	1515
Complete and duplicated BUSCOs (D)	76	76
Fragmented BUSCOs (F)	8	7
Missing BUSCOs (M)	15	16
Total BUSCO groups searched	1614	1614

Note: The lineage dataset is embryophyta_odb10.

**Table 6:** The consensus quality values of MAH1 and MAH2.

CHR	k_asm	k_total	Error rate	QV
CHR01	22610	50630337	2.35E-05	46.2877
CHR02	12507	36099562	1.82E-05	47.3903
CHR03	18092	43043340	2.21E-05	46.5509
CHR04	20933	37848568	2.91E-05	45.3586
CHR05	27201	46174067	3.10E-05	45.0845
CHR06	22142	43118196	2.70E-05	45.6809
CHR07	20602	37896126	2.86E-05	45.4333
CHR08	21345	51440721	2.18E-05	46.6068
CHR09	21992	46957838	2.47E-05	46.081
CHR10	20417	42012613	2.56E-05	45.9204
CHR11	17834	34622309	2.71E-05	45.6675
CHR12	21732	50002802	2.29E-05	46.4056
CHR13	12313	36147317	1.79E-05	47.464
CHR14	19174	42981910	2.35E-05	46.2923
CHR15	19132	37815414	2.66E-05	45.7456
CHR16	26457	45652741	3.05E-05	45.1556
CHR17	19902	41966584	2.50E-05	46.0266
CHR18	18447	37752841	2.57E-05	45.8968
CHR19	20139	51149365	2.07E-05	46.8347
CHR20	22081	47014184	2.47E-05	46.0687
CHR21	23517	45688708	2.71E-05	45.6707
CHR22	15233	34027391	2.36E-05	46.277
MAH1	225675	469843677	2.53E-05	45.9712
MAH2	218127	470199257	2.44E-05	46.1223

### Repeat and gene annotation

The extensive *de novo* TE annotator (EDTA)^35^ was used to fully screen and group repeat elements. Briefly, a *de novo* repeat library constructed by RepeatModeler v2.0.1^36^ was imported to RepeatMasker v4.1.1 (http://repeatmasker.org/) to predict repeats. Then, Repbase^37^ was introduced to predict homology repeats in RepeatMasker. In total we identified 235.46 Mb (50.11%) and 234.61 Mb (49.90%) repetitive sequences in MAH1 and MAH2, respectively. Among these, long terminal repeats (LTR) that accounted for 36.61% in MAH1 and 34.19% in MAH2 were the most abundant repeat elements (Supplementary Table S1). These results were comparable with the findings in the previous T2T DH genome version (Repeat elements: 52.62%; LTR: 34.85%)^13^.

Standard MAKER3 v3.01.03^38^ pipeline was used to annotate genes. All high-confidence protein sequences in swiss-prot^39^ database were imported for homology prediction. Transcripts from the 4 tissues, including root, leaf, flower and fruit, were used for gene prediction. Then AUGUSTUS v3.3.2 and SNAP v20131129 were used to train the ab-initio gene models. Finally, the MAKER3 pipeline was run again to obtain high-quality gene annotations. Functional characterization of the predicted coding genes was performed using eggNOG-mapper v2^40^ based on the eggNOG v5.0 database^41^. A total of 40,889 and 38,269 protein-coding genes were annotated in MAH1 and MAH2, respectively. The total lengths of protein-coding genes were 148.54 Mb and 144.95 Mb, respectively. Average lengths of genes were 3.63 kb and 3.79 kb. Based on the eggNOG-Mapper results, 59,143 (74.72%) genes were functionally annotated (Table 7). Besides, BUSCO scores of protein-coding genes in MAH1 and MAH2 were up to 89.41% and 90.27% (Table 8).

**Table 7:** Statistics of protein-coding genes in MAH1 and MAH2.

	MAH1	MAH2
Number of protein coding genes	40,889	38,269
Total length of protein coding gene (bp)	148,543,347	144,954,069
Average length of protein coding gene (bp)	3,632	3,787
Total exon length (bp)	48,236,300	49,463,483
Average length of exon (bp)	264	272
Genes with one more exon	28,286	25,849
Genes with GO terms	59,143

**Table 8:** Summary of BUSCO analysis of protein-coding genes in MAH1 (C: 89.41%) and MAH2 (C: 90.27%).

	MAH1	MAH2
Complete BUSCOs (C)	1443	1457
Complete and single-copy BUSCOs (S)	1374	1385
Complete and duplicated BUSCOs (D)	69	72
Fragmented BUSCOs (F)	75	74
Missing BUSCOs (M)	96	83
Total BUSCO groups searched	1614	1614

Note: The lineage dataset is embryophyte_odb10.

### Identification of telomeres and centromeres

TIDK v0.2.1 (https://github.com/tolkit/telomeric-identifier) was used to find telomeres. In total 36 telomeres were found (Table 9). Plant centromeric regions are generally characterized by the presence of short tandem repeats that are highly enriched in these regions^42^, accompanied by a collapse in the density of LTR elements such as *Copia*. By identifying these distinctive features, centromeric regions can be located. We predicted centromeric regions according to the workflow in Shi *et al*.^43^, which employed the above approach. Using Tandem Repeats Finder v4.09^44^ with the parameters: trf genomes.fa 2 7 7 80 10 50 500 -f -d -m, we screened 183 bp, 148 bp, 124 bp, 125 bp, and 191 bp tandem repeat units as candidates based on sorted results and IGV results (Supplementary Table S2, Supplementary Figure S3). The centromeric regions were defined according to the density of 183 bp tandem repeat unit, which was the highest enriched centromeric repeat unit. Finally, all centromeric regions have been captured successfully (Table 10, Supplementary Figure S3).

**Table 9:** Summary of telomere information of *Musa acuminata* ssp. *malaccensis* genome.

CHR	Left Start	Left End	Left Length	Right Start	Right End	Right Length
CHR1	1	13,811	13,811	50,618,246	50,630,355	12,109
CHR2	NA	NA	NA	36,081,437	36,099,580	18,143
CHR3	1	11,011	11,011	43,033,809	43,043,358	9,549
CHR4	1	11,620	11,620	NA	NA	NA
CHR5	NA	NA	NA	NA	NA	NA
CHR6	1	16,814	16,814	NA	NA	NA
CHR7	1	16,051	16,051	37,869,370	37,896,144	26,774
CHR8	1	18,389	18,389	51,435,804	51,440,739	4,935
CHR9	1	10,157	10,157	46,918,312	46,957,856	39,544
CHR10	1	5,537	5,537	41,999,867	42,012,631	12,764
CHR11	1	11,515	11,515	34,613,922	34,622,327	8,405
CHR12	1	12,705	12,705	49,988,939	50,002,820	13,881
CHR13	1	10,262	10,262	36,111,649	36,147,335	35,686
CHR14	1	17,885	17,885	42,973,945	42,981,928	7,983
CHR15	1	9,436	9,436	NA	NA	NA
CHR16	NA	NA	NA	NA	NA	NA
CHR17	1	16,814	16,814	41,892,879	41,966,602	73,723
CHR18	1	5,264	5,264	37,711,352	37,752,859	41,507
CHR19	1	12,873	12,873	51,144,443	51,149,383	4,940
CHR20	1	7,546	7,546	46,984,301	47,014,202	29,901
CHR21	1	14,084	14,084	45,671,080	45,688,726	17,646
CHR22	1	11,515	11,515	33,998,426	34,027,409	28,983

**Table 10:** Summary of centromere information of *Musa acuminata* ssp. *malaccensis* genome.

CHR	start	end	length	start_trf_id	end_trf_id
CHR01	34,370,592	38,397,126	4,026,534	TRF_12565	TRF_14496
CHR02	7,524,984	11,726,285	4,201,301	TRF_23444	TRF_26360
CHR03	21,803,558	22,962,260	1,158,702	TRF_44924	TRF_45373
CHR04	20,301,989	21,676,450	1,374,461	TRF_61392	TRF_62137
CHR05	24,142,990	25,898,645	1,755,655	TRF_78752	TRF_79866
CHR06	23,280,519	23,708,701	428,182	TRF_96837	TRF_97118
CHR07	20,753,904	22,245,710	1,491,806	TRF_113137	TRF_114095
CHR08	21,057,435	22,701,427	1,643,992	TRF_128874	TRF_129777
CHR09	24,616,864	28,481,467	3,864,603	TRF_152152	TRF_154884
CHR10	17,354,345	18,650,231	1,295,886	TRF_178427	TRF_179172
CHR11	15,992,185	17,570,998	1,578,813	TRF_193271	TRF_194294
CHR12	34,320,878	36,937,480	2,616,602	TRF_214097	TRF_215243
CHR13	7,460,959	11,858,861	4,397,902	TRF_224519	TRF_227526
CHR14	21,657,257	22,734,720	1,077,463	TRF_246003	TRF_246434
CHR15	20,157,001	21,828,979	1,671,978	TRF_262341	TRF_263283
CHR16	22,536,071	24,398,251	1,862,180	TRF_278747	TRF_280033
CHR17	21,596,438	23,729,965	2,133,527	TRF_296609	TRF_297937
CHR18	20,698,855	21,941,522	1,242,667	TRF_313322	TRF_314065
CHR19	20,950,265	23,593,891	2,643,626	TRF_328834	TRF_330287
CHR20	24,652,418	27,307,919	2,655,501	TRF_352211	TRF_354226
CHR21	18,468,410	18,926,218	457,808	TRF_383621	TRF_383906
CHR22	16,235,391	17,087,235	851,844	TRF_400050	TRF_400648

### Characterization of a reciprocal translocation in *Musa acuminata*

**Fig. 5:**
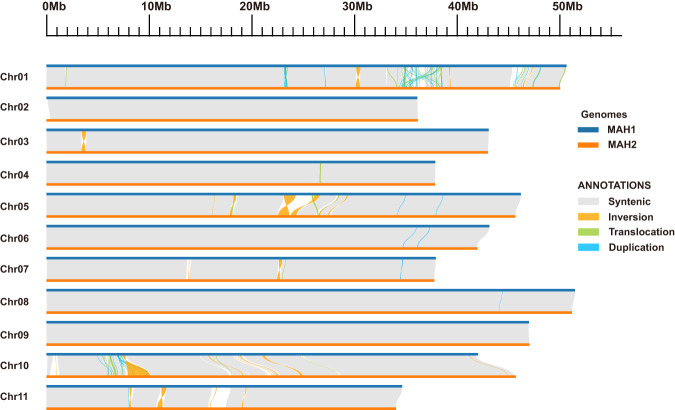
The sequence collinearity and structural variants between MAH1 and MAH2.

Nucmer v4.0.0rc1^45^ was used to obtain the syntenic relationship between MAH1 and MAH2 with default parameters. Then the delta-filter was launched with parameters ‘-i 90 -l 15000’. In the same way, our haplotype-resolved assembly was aligned against MAv4 using nucmer. Mummerplot command was used to generate the dot plots (Supplementary Figure S4). Syri v1.6.3^46^ with default parameters was used for identifying structural variants between MAH1 and MAH2 (Fig. 5). Overall, 47 translocations with a cumulative size of 2.70 Mb (~0.57%), 23 inversions with a cumulative size of 11.30 Mb (~2.40%), and 53 duplications with a cumulative size of 1.33 Mb (~0.28%) were defined. These structural variants were generally heterozygous, representing more complete genetic information compared with the double-haploid MAv4 genome.

**Fig. 6:**
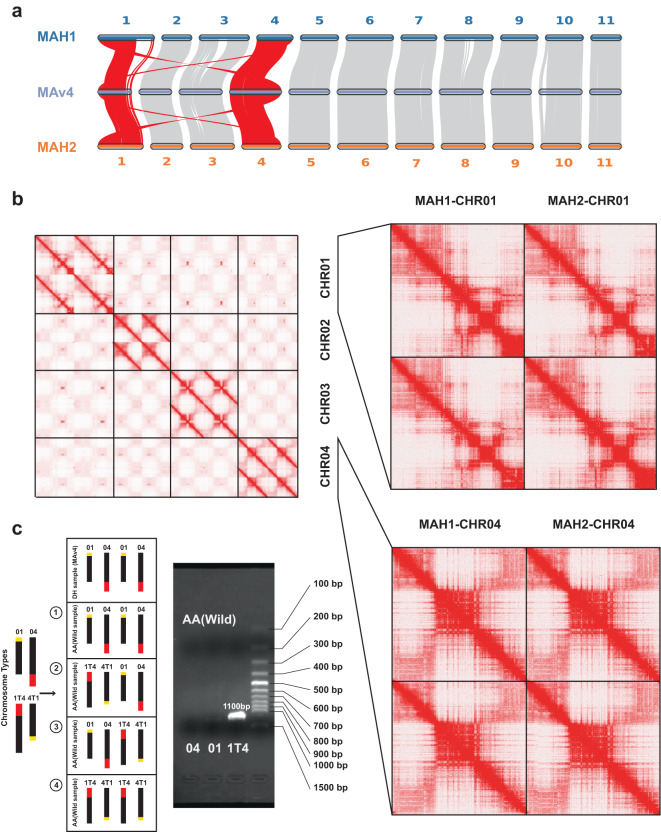
The large reciprocal translocation between chromosome 01 and chromosome 04. (**a**) Genomic synteny between DH-Pahang (MAv4) and wild *M. acuminata*. (**b**) Hi-C heatmap of chromosome 01-04. (**c**) The possible chromosome structures in wild *M. acuminata*. The targeted PCR result suggests that homozygous reciprocal translocations (only 1T4) exist in our sample, consistent with our whole genome alignment results in sequence and gene levels.

MCscan tools^47^ were used to search for the syntenic relationships between the two haploid assemblies and MAv4 at the gene level. Briefly, ‘jcvi.compara.catalog’ module with ‘--cscore = 0.99’ and ‘jcvi.compara.synteny’ module with ‘--minspan = 30’ were used to build the syntenic regions; then, syntenic relationships were visualized by ‘jcvi.graphics.karyotype’ module. Besides, potential structural variants and heterozygous regions were shown in Supplementary Figure S5. A reciprocal translocation involving 3 Mb and 10 Mb from chromosome 01 and 04 was identified (Fig. 6a). These reciprocal translocation gene blocks were located in the translocated regions identified in whole genome alignment results (Supplementary Figure S4C,D). The 10-Mb segment from 261,650 to 10,745,936 bp in chromosome 01 of MAH1 was linked to 44,882,868 to 34,419,170 bp in chromosome 04 of MAv4 (Supplementary Figure S5). The 3-Mb segment from 34,734,628 to 37,810,715 bp in chromosome 04 of MAH1 was linked to 122,362 to 3,101,126 bp in chromosome 01 of MAv4. The reciprocal translocation between MAH2 and MAv4 was located in the similar genomic regions. The huge difference in chromosome length in chromosome 01 and chromosome 04 between MAH1/2 and MAv4 was also derived from this reciprocal translocation, while other chromosome lengths and genomic total lengths were comparable (Table 3).

We further performed GO enrichments based on the extracted genes located in the translocated regions using TBtools v1.108^48^. The genes in the 10-Mb segment of MAH1 were not significantly enriched in any biological process, while those on the 3-Mb segment were enriched in several pathways associated with flower development (Supplementary Table S3), including anther development (GO:0048653), stamen development (GO:0048443), regulation of flower development (GO:0009909), and floral whorl development (GO:0048438). For further validation, we used nucleotide BLAST tools in National Center for Biotechnology Information (NCBI) with default parameters to align identified genes to non-redundant database, and checked gene functions manually.

## Data Records

All raw sequencing reads have been deposited in the National Center for Biotechnology Information (NCBI) under BioProject ID PRJNA962682, and the National Genomics Data Center (NGDC) under BioProject ID PRJCA018611. The PacBio HiFi, Nanopore, Hi-C, Illumina sequencing reads have been deposited in the NCBI Sequence Read Archive database with accession group numbers SRP435127^49^. Genome assembly is available from GenBank with accession number GCA_030219345.1^50^. The genome annotation files have been submitted to the online open access repository Figshare database^51^, including a high-quality reference genome that we constructed for guidance.

## Technical Validation

### Manual correction for chromosome scaffolding

For constructing a high-quality reference genome, we used Juicebox to manually correct the reference genome based on Hi-C alignments (Supplementary Figure S1). Finally, 471.04 Mb (95.83%) contigs were anchored to 11 pseudo-chromosomes. Then, we started to orient, sort and group our haplotype-resolved genome based on this high-quality reference genome. We also used Juicebox to manually correct the haplotype-resolved genome based on Hi-C alignments. In total, 469.83 Mb (93.82%) and 470.21 Mb (97.08%) contigs were anchored to 11 chromosome pairs, respectively. We further examined the Hi-C alignments in chromosome 01 and chromosome 04 in Juicebox (Fig. 6b), and confirmed the accurate assemblies of chromosome 01 and chromosome 04. Besides, chromosome 01 consists of only one contig (Table 3), further confirming its high continuity.

### Targeted PCR confirmed the reciprocal translocation between Chr01 and Chr04

Based on the genomic syntenic analysis between our assembly and MAv4, we identified a large reciprocal translocation from chromosomes 01 and 04, corresponding to the translocation found in a previous study^9^. In that study, three pairs of primers were designed to amplify the breakpoints located along the reference and hypothesized chromosome structures, thereby showing the presence of chromosomes 01, 04, and 1T4 resulting from the translocation. Here we used the same primer pairs to perform targeted PCR to validate the chromosome structures found in our sample (Fig. 6c). DNA was extracted from leaf tissue of *M*. *acuminata* ssp. *malaccensis*. PCR was performed in 50-μL volumes containing 2.5 ng of gDNA, 1 μL of specific primers, 32 μL of distilled, deionized water, and 0.5 μL of *TaKaRa LA Taq*^®^ (Vazyme) using an Eastwin Life Science EDC810 PCR amplification system. The reaction conditions for thermal cycling were 94 °C for 5 min, followed by 35 cycles of 94 °C for 45 s, 56 °C for 45 s, and 72 °C for 60 s. Thereafter, PCR products were visualized by 2% agarose gel-electrophoresis with a 100 bp DNA ladder. Only the breakpoint of chromosome 1T4 was amplified in our studied sample, suggesting that the reciprocal translocation involving 3 and 10 Mb segments from chromosomes 01 and 04 existed in both haploid genomes of the *M. acuminata* sample (Fig. 6c). This finding was consistent with our whole genome alignment results in sequence and gene levels.

### Supplementary information

Supplementary Information

## Data Availability

No special code was used for this study. All software mentioned in methods could be found in the community. If no detail parameters were mentioned for the software, default parameters were used as suggested by the developer.

## References

[1] Brozynska M, Furtado A, Henry RJ (2016). Genomics of crop wild relatives: expanding the gene pool for crop improvement. Plant Biotechnol. J..

[2] Bohra A (2022). Reap the crop wild relatives for breeding future crops. Trends Biotechnol..

[3] Castaneda-Alvarez NP (2016). Global conservation priorities for crop wild relatives. Nat. Plants.

[4] Perrier X (2011). Multidisciplinary perspectives on banana (*Musa* spp.) domestication. Proc. Natl. Acad. Sci. USA.

[5] Davey MW (2013). A draft *Musa balbisiana* genome sequence for molecular genetics in polyploid, inter- and intra-specific *Musa* hybrids. BMC Genom..

[6] Perrier X (2009). Combining biological approaches to shed light on the evolution of edible bananas. Ethnobot. Res. App..

[7] Shepherd K. *Cytogenetics Of The Genus Musa* (International Network for the Improvement of Banana and Plantain, 1999).

[8] Hippolyte I (2010). A saturated SSR/DarT linkage map of *Musa acuminata* addressing genome rearrangements among bananas. BMC Plant Biol..

[9] Martin G (2017). Evolution of the banana genome (*Musa acuminata*) is impacted by large chromosomal translocations. Mol. Biol. Evol..

[10] Dupouy M (2019). Two large reciprocal translocations characterized in the disease resistance-rich *burmannica* genetic group of *Musa acuminata*. Ann. Bot..

[11] Martin G (2020). Chromosome reciprocal translocations have accompanied subspecies evolution in bananas. Plant J..

[12] D’Hont A (2012). The banana (*Musa acuminata*) genome and the evolution of monocotyledonous plants. Nature.

[13] Belser C (2021). Telomere-to-telomere gapless chromosomes of banana using nanopore sequencing. Commun. Biol..

[14] Hu G (2022). Two divergent haplotypes from a highly heterozygous lychee genome suggest independent domestication events for early and late-maturing cultivars. Nat. Genet..

[15] Sun X (2020). Phased diploid genome assemblies and pan-genomes provide insights into the genetic history of apple domestication. Nat. Genet..

[16] Marcais G, Kingsford C (2011). A fast, lock-free approach for efficient parallel counting of occurrences of k-mers. Bioinformatics.

[17] Ranallo-Benavidez TR, Jaron KS, Schatz MC (2020). GenomeScope 2.0 and Smudgeplot for reference-free profiling of polyploid genomes. Nat. Commun..

[18] Chen S, Zhou Y, Chen Y, Gu J (2018). fastp: an ultra-fast all-in-one FASTQ preprocessor. Bioinformatics.

[19] Cheng H, Concepcion GT, Feng X, Zhang H, Li H (2021). Haplotype-resolved *de novo* assembly using phased assembly graphs with hifiasm. Nat. Methods.

[20] Alonge M (2019). RaGOO: fast and accurate reference-guided scaffolding of draft genomes. Genome Biol..

[21] Durand NC (2016). Juicer provides a one-click system for analyzing loop-resolution Hi-C experiments. Cell Syst..

[22] Dudchenko O (2017). *De novo* assembly of the *Aedes aegypti* genome using Hi-C yields chromosome-length scaffolds. Science.

[23] Durand NC (2016). Juicebox provides a visualization system for Hi-C contact maps with unlimited zoom. Cell Syst..

[24] Hu J, Fan J, Sun Z, Liu S (2020). NextPolish: a fast and efficient genome polishing tool for long-read assembly. Bioinformatics.

[25] Li H (2018). Minimap2: pairwise alignment for nucleotide sequences. Bioinformatics.

[26] Thorvaldsdottir H, Robinson JT, Mesirov JP (2013). Integrative Genomics Viewer (IGV): high-performance genomics data visualization and exploration. Brief. Bioinform..

[27] Gurevich A, Saveliev V, Vyahhi N, Tesler G (2013). QUAST: quality assessment tool for genome assemblies. Bioinformatics.

[28] Krzywinski M (2009). Circos: an information aesthetic for comparative genomics. Genome Res..

[29] Li H, Durbin R (2009). Fast and accurate short read alignment with Burrows-Wheeler transform. Bioinformatics.

[30] Kim D, Paggi JM, Park C, Bennett C, Salzberg SL (2019). Graph-based genome alignment and genotyping with HISAT2 and HISAT-genotype. Nat. Biotechnol..

[31] Barnett DW, Garrison EK, Quinlan AR, Stromberg MP, Marth GT (2011). BamTools: a C++ API and toolkit for analyzing and managing BAM files. Bioinformatics.

[32] Ou S, Jiang N (2018). LTR_retriever: a highly accurate and sensitive program for identification of long terminal repeat retrotransposons. Plant Physiol..

[33] Manni M, Berkeley MR, Seppey M, Simao FA, Zdobnov EM (2021). BUSCO update: novel and streamlined workflows along with broader and deeper phylogenetic coverage for scoring of eukaryotic, prokaryotic, and viral genomes. Mol. Biol. Evol..

[34] Rhie A, Walenz BP, Koren S, Phillippy AM (2020). Merqury: reference-free quality, completeness, and phasing assessment for genome assemblies. Genome biol..

[35] Ou S (2019). Benchmarking transposable element annotation methods for creation of a streamlined, comprehensive pipeline. Genome Biol..

[36] Flynn JM (2020). RepeatModeler2 for automated genomic discovery of transposable element families. Proc. Natl. Acad. Sci. USA.

[37] Bao W, Kojima KK, Kohany O (2015). Repbase Update, a database of repetitive elements in eukaryotic genomes. Mob. DNA.

[38] Campbell MS, Holt C, Moore B, Yandell M (2014). Genome annotation and curation using MAKER and MAKER-P. Curr Protoc Bioinformatics.

[39] Bairoch A, Apweiler R (2000). The SWISS-PROT protein sequence database and its supplement TrEMBL in 2000. Nucleic Acids Res..

[40] Cantalapiedra CP, Hernandez-Plaza A, Letunic I, Bork P, Huerta-Cepas J (2021). eggNOG-mapper v2: functional annotation, orthology assignments, and domain prediction at the metagenomic scale. Mol. Biol. Evol..

[41] Huerta-Cepas J (2019). eggNOG 5.0: a hierarchical, functionally and phylogenetically annotated orthology resource based on 5090 organisms and 2502 viruses. Nucleic Acids Res..

[42] Melters DP (2013). Comparative analysis of tandem repeats from hundreds of species reveals unique insights into centromere evolution. Genome Biol..

[43] Shi X (2023). The complete reference genome for grapevine (*Vitis vinifera* L.) genetics and breeding. Hortic. Res..

[44] Benson G (1999). Tandem repeats finder: a program to analyze DNA sequences. Nucleic Acids Res..

[45] Marcais G (2018). MUMmer4: A fast and versatile genome alignment system. PLoS Comput. Biol..

[46] Goel M, Sun H, Jiao WB, Schneeberger K (2019). SyRI: finding genomic rearrangements and local sequence differences from whole-genome assemblies. Genome Biol..

[47] Tang H (2008). Synteny and collinearity in plant genomes. Science.

[48] Chen C (2020). TBtools: an integrative toolkit developed for interactive analyses of big biological data. Mol. Plant.

[49] (2023). NCBI Sequence Read Archive.

[50] Liu X (2023). GenBank.

[51] Liu X (2023). Figshare.

