# Supplementary material for: The phased telomere-to-telomere reference genome of Musa acuminata, a main contributor to banana cultivars
Source: Sci Data. 2023 Sep 16;10:631. doi: 10.1038/s41597-023-02546-9 (PMC10505225; doi:10.1038/s41597-023-02546-9)
Supplement: Supplementary file 1 — Supplementary Information [file 41597_2023_2546_MOESM1_ESM.pdf]

Supplementary information for:

The phased telomere-to-telomere reference genome of *Musa acuminata*, a main contributor to banana cultivars

Xin Liu, Rida Arshad, Xu Wang, Wei-Ming Li, Yongfeng Zhou, Xue-Jun Ge, Hui-Run Huang

Supplementary	Title	Pages
Figure S1	The Hi-C heatmap of the unphased MA reference genome.	2
Figure S2	The consensus quality values (QV) of MAH1 and MAH2.	3
Figure S3	The potential centromeric regions of *Musa acuminata* ssp. *malaccensis* based on the density of the 183bp tandem repeat units.	4
Figure S4	The Mummerplot of MAH1, MAH2 and MAv4.	5
Figure S5	The structural variants between MAH1, MAH2 and MAv4.	6
Table S1	Statistics of repeats content in MAH1 and MAH2.	7-8
Table S2	Summary of different repeat units on centromeres in all chromosomes.	9
Table S3	The GO enrichment results of the genes located in the 3-Mb translocated region (34,734,628 - 37,827,340 bp of chromosome 04) in MAH1.	10-12

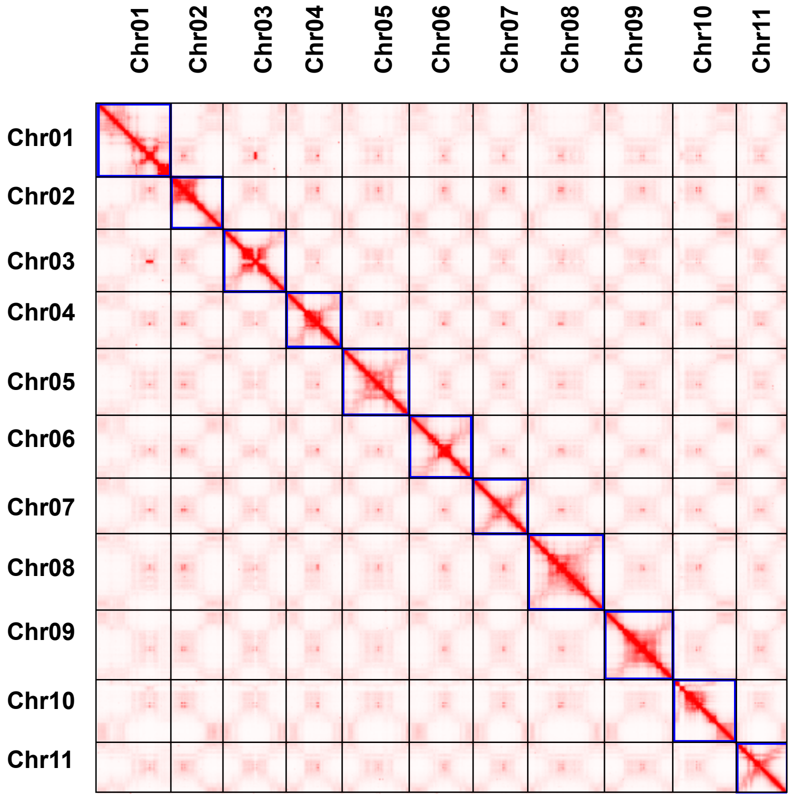

Supplementary Figure S1. The Hi-C heatmap of the unphased MA reference genome.

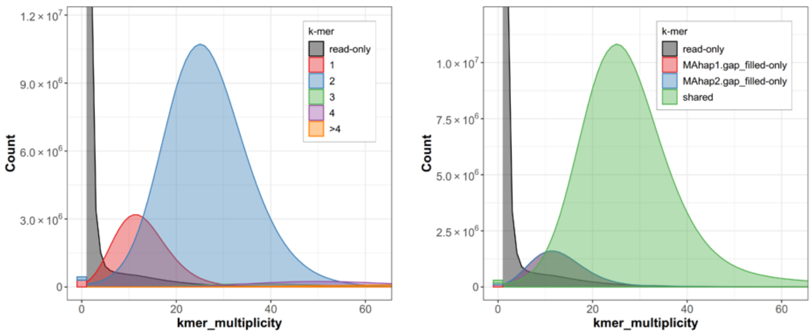

Supplementary Figure S2. The consensus quality values (QV) of MAH1 and MAH2.

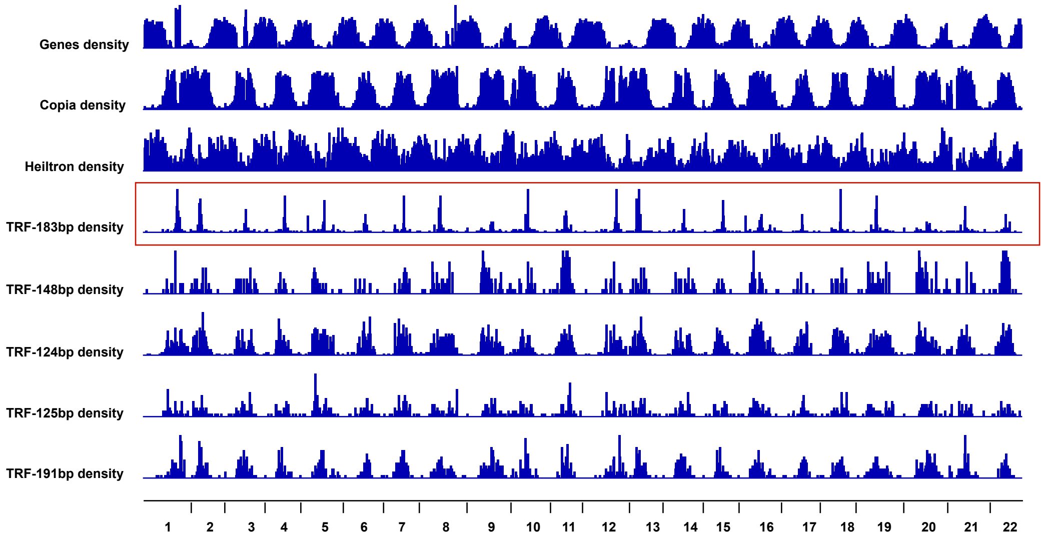

Supplementary Figure S3. The potential centromeric regions of *Musa acuminata* ssp. *malaccensis* based on the density of the 183bp tandem repeat units.

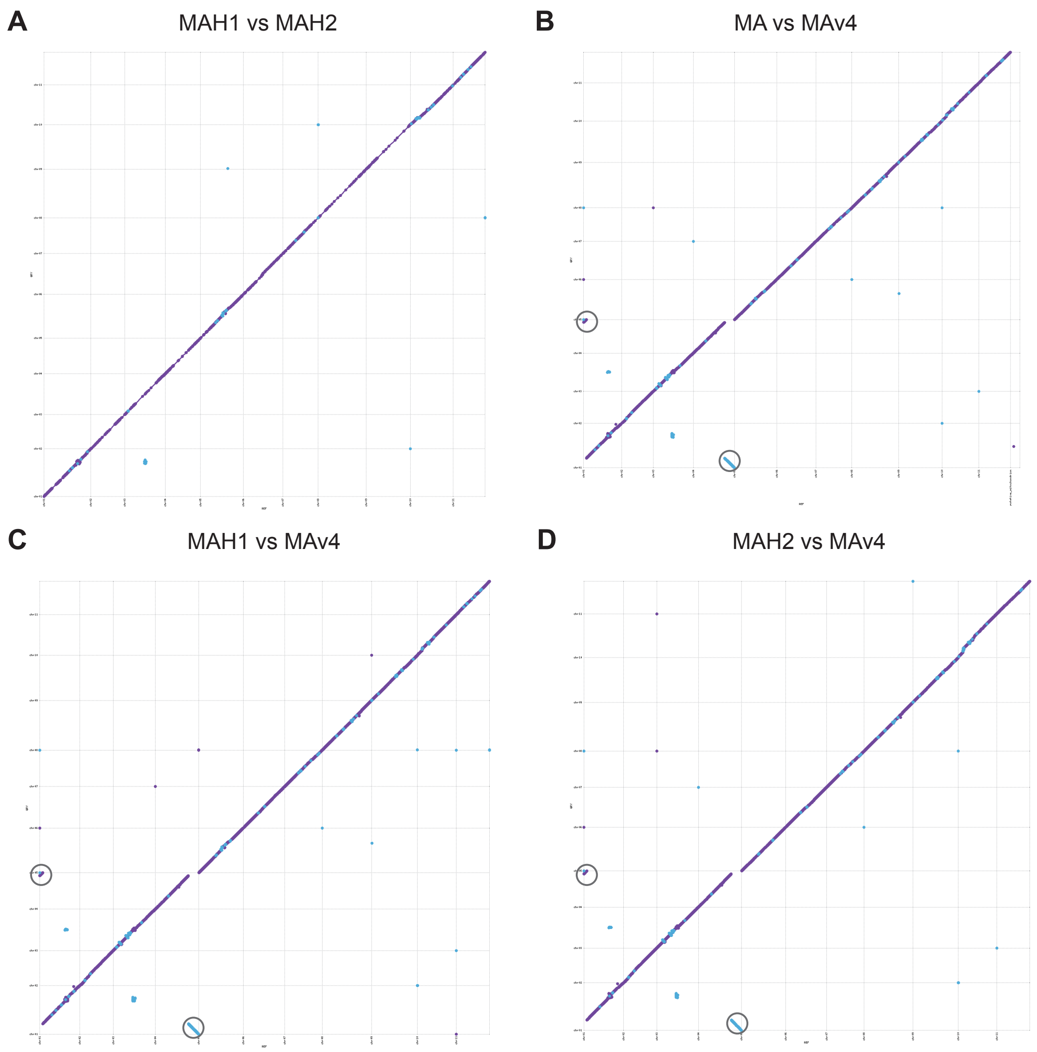

Supplementary Figure S4. The Mummerplot of MAH1, MAH2 and MAv4. The phased subgenomes show high consistency (A), while a translocation was shown in chromosome 01 and chromosome 04 between our phased or unphased genomes and MAv4 (B, C and D).

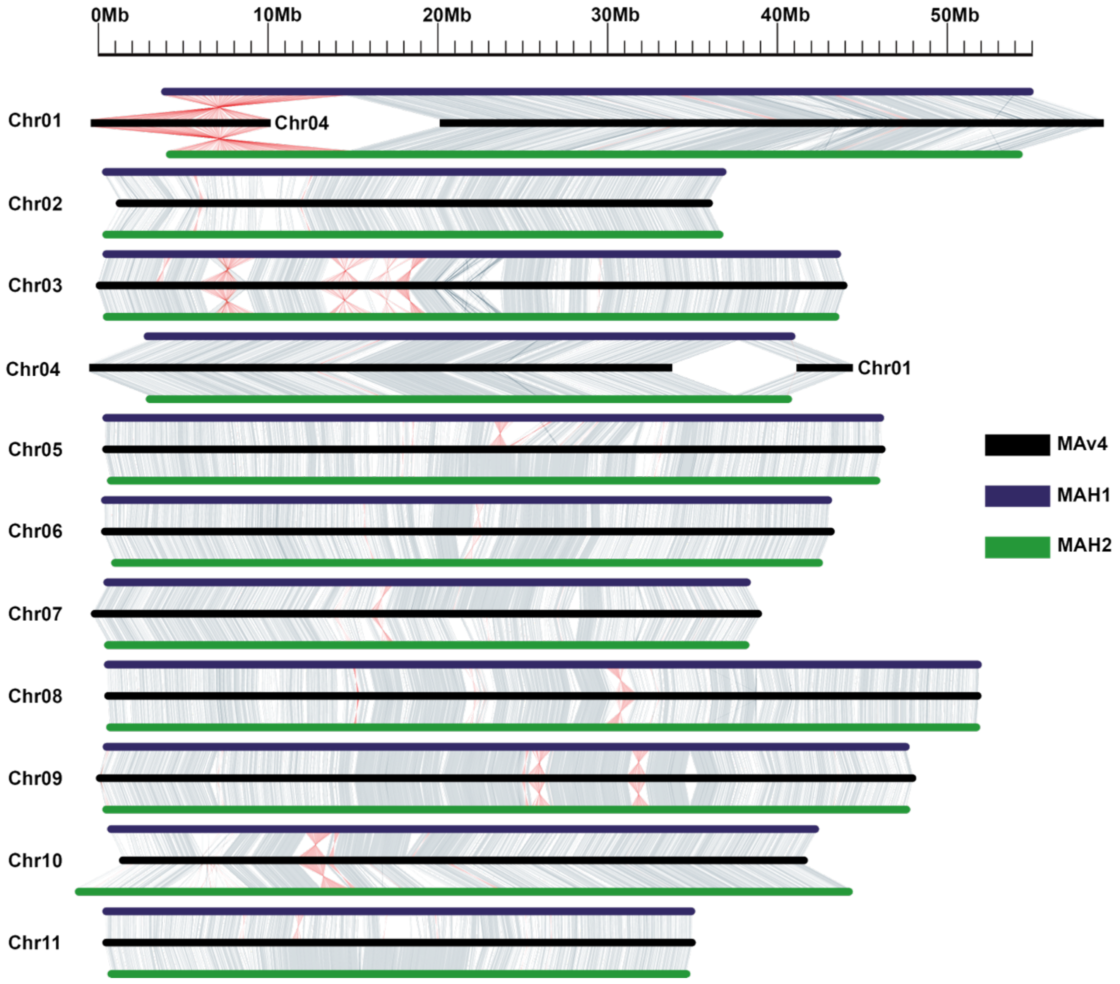

Supplementary Figure S5. The structural variants between MAH1, MAH2 and MAv4.

Supplementary Table S1. Statistics of repeats content in MAH1 and MAH2

Genome name:		MAH1		
Chromosomes number:		11		
Total length (bp):		469843875		
GC level:		0.3912		
Bases masked:		235459590 bp (50.11%)		

**Class**		**Count**	**bpMasked**	**%masked**
LTR		--	--	--
	Copia	142871	143678256	30.58%
	Gypsy	47959	28308096	6.03%
	unknown	47075	24396053	5.19%
TIR		--	--	--
	CACTA	5285	1636100	0.35%
	Mutator	43580	20588170	4.38%
	PIF_Harbinger	1459	567348	0.12%
	Tc1_Mariner	1755	387440	0.08%
	hAT	10731	4084895	0.87%
nonLTR		--	--	--
	LINE_element	255	112054	0.02%
nonTIR		--	--	--
	helitron	10060	3190605	0.68%
repeat_region		25438	8510573	1.81%
Total		336468	235459590	50.11%

Genome name:	MAH2
Chromosomes number:	11
Total length (bp):	470199455
GC level:	0.392
Bases masked :	234610719 bp (49.90 %)

**Class**		**Count**	**bpMasked**	**%masked**
LTR		--	--	--
	Copia	137921	131788092	28.03%
	Gypsy	36843	28984780	6.16%
	unknown	60198	25579893	5.44%
TIR		--	--	--
	CACTA	8332	2653785	0.56%
	Mutator	35418	26069401	5.54%
	PIF_Harbinger	1196	445937	0.09%
	Tc1_Mariner	1184	299030	0.06%
	hAT	11681	4358177	0.93%
nonLTR		--	--	--
	LINE_element	2378	1640147	0.35%
nonTIR		--	--	--
	helitron	7952	2532993	0.54%
repeat_region		27089	10258484	2.18%
Total		330192	234610719	49.90%

Note: The information above is the default output of EDTA with parameters -species others s -step all -evaluate 1 -sensitive 1 -anno

Supplementary Table S2. Summary of different repeat units on centromeres in all chromosomes.

chr01

units	copies
469	6220
124	403
471	166
183	163
191	163

chr02

units	copies
124	402
183	148
191	136
125	112
134	102

chr03

units	copies
469	4015
124	314.4
191	160.2
120	99.8
125	97.2

chr04

units	copies
124	300.1
183	105.9
117	102
191	99.8
125	84.8

chr05

units	copies
124	473
125	161
191	145
183	139
120	90.4

chr06

units	copies
124	392
125	112
191	94.4
186	87.9
151	82.3

chr07

units	copies
124	422.3
191	147.9
125	115.9
183	96
107	60.9

chr08

units	copies
124	547.5
427	498.5
405	359.7
191	158.8
125	136.2

chr09

units	copies
124	461
191	206
148	155
125	127
120	98

chr10

units	copies
220	4006
124	336
191	153
183	143
109	122

chr11

units	copies
124	369.8
148	145.9
191	127.6
125	122.8
183	102.5

chr12

units	copies
469	6966
124	359.8
471	236.8
191	152.8
183	145.5

chr13

units	copies
124	406
183	150
191	136
125	116
134	100

chr14

units	copies
469	3966
124	317
191	158
120	105
125	97.2

chr15

units	copies
124	296
183	101.9
191	99.8
117	97.9
125	86.8

chr16

units	copies
124	501.3
125	144
183	138.3
191	129.3
105	89.7

chr17

units	copies
124	366
191	97.7
151	85
125	84.5
186	80.7

chr18

units	copies
124	419
191	128
125	112
183	99.3
148	62.5

chr19

units	copies
124	555.6
405	359.7
427	346.5
191	164.8
125	136.5

chr20

units	copies
124	455
191	205.8
148	157.1
125	125.1
120	95.7

chr21

units	copies
220	6472
124	402
191	183
109	155
183	108

chr22

units	copies
124	370
148	146
191	94.1
125	90.4
105	68.2

Supplementary Table S3. The GO enrichment results of the genes located in the 3-Mb translocated region (34,734,628 - 37,827,340 bp of chromosome 04) in MAH1. MF, Molecular function; CC, Cellular component;BP, Biological process.

Class	GO_Name	GO_ID	GO_Level	P_value	EnrichmentScore
MF	transporter activity	GO:0005215	2	0.00639684	1.80773097
MF	monoatomic anion transmembrane transporter activity	GO:0008509	5	0.007944	2.79423559
MF	monoatomic ion transmembrane transporter activity	GO:0015075	4	0.01098306	2.04073386
MF	transmembrane transporter activity	GO:0022857	3	0.01175789	1.77277787
MF	organic anion transmembrane transporter activity	GO:0008514	4	0.01355187	3.0721962
MF	monoatomic cation transmembrane transporter activity	GO:0008324	5	0.01628308	2.30414715
MF	inorganic molecular entity transmembrane transporter activity	GO:0015318	4	0.0238958	1.89899506
MF	inorganic cation transmembrane transporter activity	GO:0022890	5	0.03115188	2.17329435
CC	obsolete intrinsic component of membrane	GO:0031224	0	2.50E-04	2.31670098
CC	nuclear body	GO:0016604	3	0.0015778	4.12173794
CC	obsolete extracellular region part	GO:0044421	0	0.00423464	4.7047849
CC	chloroplast	GO:0009507	7	0.00470173	1.55392324
CC	plastid	GO:0009536	6	0.0052209	1.53039472
CC	nuclear speck	GO:0016607	4	0.00701182	4.17293965
CC	chloroplast membrane	GO:0031969	6	0.0105267	3.77864614
CC	plastid membrane	GO:0042170	5	0.01340196	2.25829675
CC	organelle membrane	GO:0031090	4	0.01507675	1.49965019
CC	obsolete membrane part	GO:0044425	0	0.01664485	1.55002603
CC	thylakoid	GO:0009579	6	0.02380688	2.05960541
CC	microbody	GO:0042579	6	0.02402427	2.7035947
CC	peroxisome	GO:0005777	7	0.02402427	2.7035947

CC	bounding membrane of organelle	GO:0098588	5	0.02701417	1.55519279
CC	plastid envelope	GO:0009526	5	0.02771339	1.7610571
CC	chloroplast envelope	GO:0009941	6	0.03934272	1.82340886
CC	chloroplast thylakoid	GO:0009534	8	0.04408488	2.03127221
CC	obsolete chloroplast part	GO:0044434	0	0.04510026	1.50958164
CC	plastid thylakoid	GO:0031976	7	0.04523039	2.0205813
CC	vacuolar membrane	GO:0005774	6	0.04633399	1.77437602
CC	plant-type vacuole	GO:0000325	7	0.04679628	2.55259606
CC	obsolete cytoplasmic part	GO:0044444	0	0.04947538	1.13796475
BP	anther development	GO:0048653	4	0.00383367	4.8162963
BP	organonitrogen compound catabolic process	GO:1901565	5	0.00453555	2.0659716
BP	protein catabolic process	GO:0030163	6	0.00588219	2.13718378
BP	monoatomic anion transmembrane transport	GO:0098656	7	0.0060345	2.71765935
BP	photosynthesis	GO:0015979	4	0.00626139	3.6349406
BP	post-embryonic development	GO:0009791	3	0.01672247	1.45055512
BP	monoatomic anion transport	GO:0006820	6	0.01728145	2.28742304
BP	organic substance catabolic process	GO:1901575	4	0.02028085	1.60161872
BP	monoatomic ion transmembrane transport	GO:0034220	6	0.02036939	1.83477954
BP	cellular response to chemical stress	GO:0062197	5	0.02135026	2.77863248
BP	stamen development	GO:0048443	6	0.02810038	2.93676603
BP	androecium development	GO:0048466	5	0.02810038	2.93676603
BP	regulation of flower development	GO:0009909	7	0.02823929	2.6034034
BP	proteolysis involved in protein catabolic process	GO:0051603	7	0.02864777	1.91953838
BP	monoatomic cation transmembrane transport	GO:0098655	7	0.03258005	2.04465409
BP	transmembrane transport	GO:0055085	5	0.0329954	1.5656053

BP	catabolic process	GO:0009056	3	0.03474745	1.49658924
BP	floral whorl development	GO:0048438	4	0.03977634	2.21075896
BP	post-embryonic plant morphogenesis	GO:0090698	4	0.04113594	2.06044761
BP	macromolecule catabolic process	GO:0009057	5	0.04141326	1.6308001
BP	organic anion transport	GO:0015711	6	0.04535028	2.32110665
BP	seedling development	GO:0090351	4	0.0483355	2.28440931
BP	protein ubiquitination	GO:0016567	9	0.04870786	1.82090597
